# A randomised trial evaluating bevacizumab as adjuvant therapy following resection of AJCC stage IIB, IIC and III cutaneous melanoma: an update

**DOI:** 10.3332/ecancer.2008.108

**Published:** 2008-11-12

**Authors:** S Biswas, J Wrigley, C East, A Hern, A Marshall, J Dunn, P Lorigan, M Middleton, P Corrie

**Affiliations:** 1 Division of Oncology, Oncology Centre, Addenbrooke’s Hospital, Cambridge, CB0 2QQ, UK; 2 Warwick Medical School Clinical Trials Unit, University of Warwick, UK; 3 Department of Medical Oncology, Christie Hospital, Manchester, UK; 4 CR UK Medical Oncology Unit, Oxford Cancer Centre, Churchill Hospital, Oxford, UK

## Abstract

At present, there are no standard therapies for the adjuvant treatment of malignant melanoma. Patients with primary tumours with a high-Breslow thickness (stages IIB and IIC) or with resected loco-regional nodal disease (stage III) are at high risk of developing metastasis and subsequent disease-related death. Given this, it is important that novel therapies are investigated in the adjuvant melanoma setting. Since angiogenesis is essential for primary tumour growth and the development of metastasis, anti-angiogenic agents are attractive potential therapeutic candidates for clinical trials in the adjuvant setting. Therefore, we initiated a phase II trial in resected high-risk cutaneous melanoma, assessing the efficacy of bevacizumab versus observation.

In the interim safety data analysis, we demonstrate that bevacizumab is a safe therapy in the adjuvant melanoma setting with no apparent increase in the surgical complication rate after either primary tumour resection and/or loco-regional lymphadenectomy.

## Introduction

Many prospective randomised trials of adjuvant therapy, particularly using cytokine immunotherapies [1,2], have been performed in patients after resection of cutaneous melanoma. However, to date, no treatment has convincingly improved overall disease survival.

Since angiogenesis is fundamental to the development and growth of malignant melanomas [3], this aspect of melanoma biology is an attractive candidate for therapeutic intervention. *In vitro* pre-clinical studies either using single-agent angiogenic inhibitors or in combination with growth factor pathway tyrosine kinase inhibitors (TKIs) have demonstrated cytocidal effects in melanoma and epithelial cancer cells [4,5].

Vascular endothelial growth factor (VEGF) isoforms are the most important pro-angiogenic ligands in tumour biology [6]. Based on pre-clinical studies, VEGF isoforms, particularly VEGF-A, are relevant targets in melanoma. Immunohistochemical studies suggest that VEGF-A is expressed in around two-thirds of primary melanomas [7], and high expression levels of VEGF-A are associated with vertical phase tumour growth and disease progression to both loco-regional and distant sites [8]. Notably, loco-regional nodal metastasis seem to differentially express specific VEGF-A splice variants and express higher levels of VEGF121 and VEGF165 when compared to distant metastatic sites [8].

Bevacizumab (Avastin, F Hoffman-La Roche Ltd) is a recombinant humanized IgG1 monoclonal antibody to pan-VEGF isoforms. To date, bevacizumab has been investigated in the metastatic first- and second-line settings for colorectal cancer [9,10], as well as anthracycline-refractory breast cancer [11] and in combination with interferon-alpha-2a in clear-cell renal cancer [12]. These studies have confirmed bevacizumab to have an acceptable toxicity profile, with fatigue, hypertension and proteinuria being the commonest toxicities. The results of a phase III trial investigating the utility and toxicity profile of bevacizumab in the adjuvant management of resected colorectal cancer, after systemic chemotherapy, is currently awaited [13]. This trial could also be informative as to whether bevacizumab increases the incidence of intermediate- to long-term post-surgical complications, after adjuvant chemotherapy.

A series of small phase II studies in metastatic melanoma suggests that bevacizumab also has activity in this disease [14]. Since angiogenesis is fundamental in the development of systemic metastasis, anti-angiogenic therapy after complete resection of cutaneous melanoma is therefore an attractive candidate for investigation. Therefore, at our cancer centre, we have initiated a phase III trial investigating bevacizumab versus observation in patients with fully resected high-risk cutaneous melanoma.

## Methods: AVAST-M trial design

The UK AVAST-M Phase III Multi-Centre Prospective Clinical Trial, funded by Cancer Research UK, randomises melanoma patients at high risk of recurrence to receive bevacizumab 7.5 mg/kg IV three-week administered for one year, or to standard observation.

Overall survival will be compared in 1320 patients, to identify an 8% difference in overall survival at five years (80% power, 5% significance) as the primary end point.

## Trial objectives

**Primary objective**
- Overall survival

**Secondary objectives**
- Disease-free interval
- Distant metastasis-free interval
- Safety and toxicity
- Quality of life (QoL)

**Tertiary objectives**
- Identification of markers of angiogenesis in peripheral blood and tumour tissue

## Trial population

**Main inclusion criteria**
- Patients with histological confirmation of completely resected American Joint Commission on Cancer (AJCC) stage IIB (T3bN0M0 and T4aN0M0), IIC (T4bN0M0) and III (TxN1-3M0) cutaneous melanoma.
- Patients may or may not have undergone sentinel lymph node dissection and/or elective lymph node dissection.
- Patients must be randomised within 12 weeks of completing surgery (wide local excision or lymphadenectomy).

**Figure:**
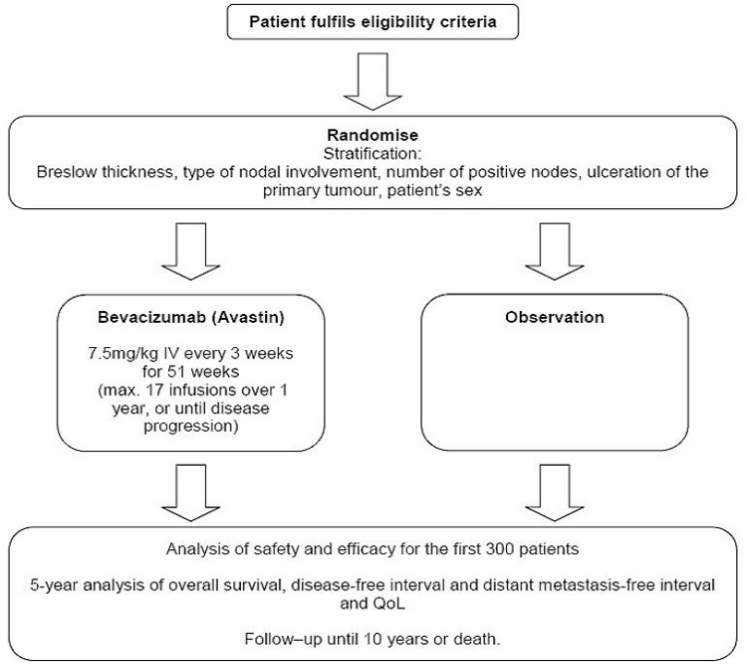

**Main exclusion criteria**
- Any evidence of distant or non-regional lymph node metastases.
- Evidence of CNS metastases, even if previously treated.
- Incomplete surgical resection of the disease.
- Prior chemotherapy, immunotherapy or hormonal therapy for melanoma within 12 weeks of randomisation.
- History or evidence of any disorder with increased risk of bleeding.
- Uncontrolled hypertension.

**Main trial assessments**
- CT/MRI scans of head, chest, abdomen and pelvis (only at baseline and subsequently if clinically indicated).
- Chest x-rays.
- Protein urinalysis.
- Haematology and clinical chemistry tests.
- Quality-of-life EORTC QLQC30 questionnaire.
- Blood and tissue for biomarker studies.

## Current status of AVAST-M

The AVAST-M trial opened to recruitment in the UK in July 2007.

As of 31 July 2008, 156 patients had been recruited: 76 on treatment arm, 80 on observation. To date, nine patients have been withdrawn from treatment: three due to distant recurrence, two due to toxicity, one due to local recurrence and three patients withdrew consent. There have been no patient deaths to 31 July 2008.

## Patient recruitment of AVAST-M

**Figure:**
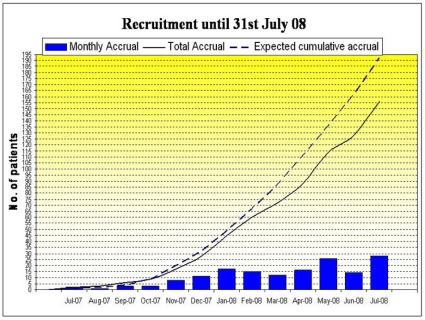


## Toxicities of AVAST-M

Six serious adverse episodes (SAEs), including one suspected unexpected serious adverse reaction (SUSAR), have been reported to date; two for the observation arm and four for the treatment arm.

In the observation arm, one patient had a grade 3 skin infection and was hospitalized for antibiotic therapy, and another patient had spinal cord compression caused by disseminated disease.

In the treatment arm, one patient who was previously normotensive, had bevacizumab-induced grade 3 hypertension. Two other patients had toxicities not related to bevacizumab. One patient, with a previous history of hiatus hernia and dyspeptic symptoms, had grade 3 upper abdominal pain in the absence of gastro-intestinal perforation. The second patient had an infected seroma at the primary surgical site, which was subsequently found to be due to a local recurrence.

Importantly, a SUSAR was identified in the bevacizumab arm. A 48-year old man presented with right eye pain and loss of vision on day 11 after his first cycle of bevacizumab; by day 18, he had lost all sight in that eye. Ophthalmology review on day 19 suggested optic neuritis. No other neurological symptoms were present and there was no history of demyelinating diseases. CT and MRI of head were normal. Posterior leukoencephalopathy syndrome was not identified, and no cases of bevacizumab-related optic neuritis have been previously reported in the literature. The patient was withdrawn from treatment and has regained some vision. Long-term ophthalmology follow-up has been arranged for this patient.

## Discussion

A 12-month safety review of the AVAST-M trial suggests that adjuvant bevacizumab is very well tolerated in patients with completely resected cutaneous malignant melanoma. The translational component of this trial could also potentially identify potential biomarkers of minimal residual disease, predictive markers for bevacizumab response, as well as identifying markers of disease relapse.

We would like to increase the profile and recruitment of patients onto this trial in the wider international oncology community. We would like to hear from interested cancer physicians if they wish to participate in AVAST-M by contacting the oncology trials team at Addenbrooke’s Hospital, Cambridge, by using either of the two following e-mail addresses: bhawna.sirohi@addenbrookes.nhs.uk or clare.east@addenbrookes.nhs.uk.
